# Diatom diversity and distribution in Madeira Island streams (Portugal)

**DOI:** 10.3897/BDJ.8.e59813

**Published:** 2020-12-16

**Authors:** Catarina Ritter, Pedro M. Raposeiro, Vítor Gonçalves

**Affiliations:** 1 CIBIO, Research Center in Biodiversity and Genetic Resources, InBIO Associate Laboratory / Faculty of Sciences and Technology, University of the Azores, Ponta Delgada, Portugal CIBIO, Research Center in Biodiversity and Genetic Resources, InBIO Associate Laboratory / Faculty of Sciences and Technology, University of the Azores Ponta Delgada Portugal

**Keywords:** Bacillariophyta, oceanic islands, freshwater systems, new records, diatom occurrences

## Abstract

**Background:**

Here, we present the data obtained from the samples collected in a field campaign during the spring of 2015 which aims for a better understanding of the diversity and distribution patterns of freshwater diatoms in Madeira Island. Following European and Portuguese standards and recommendations for routine diatom sampling and analysis, we collected samples in 40 sites, distributed in 27 permanent streams and identified the diatom species present, using general diatom floras and studies in Portuguese freshwater diatoms.

**New information:**

Little is known about the diversity and distribution of freshwater diatom assemblages from Madeira Archipelago. This study reports a survey in 40 sites in Madeira Island distributed in 27 permanent streams. A total of 965 diatom (Bacillariophyta) occurrences were recorded, belonging to 130 different taxa from 44 genera and 27 families. The families with the highest number of occurrences were Bacillariaceae (176), Achnanthidiaceae (135) and Naviculaceae (133). The two diatom endemisms, described previously in Madeira Island (Lange-Bertalot 1993), *Nitzschia macaronesica* Lange-Bertalot and *Navicula madeirensis* Lange-Bertalot, were only observed in a small number of sites, located mostly at Laurissilva forest. Sixty species are new records, not only to Madeira Island, but also to the Madeira Archipelago.

## Introduction

Diatoms (Bacillariophyta) are microscopic algae and one of the most abundant and diverse group of aquatic, pigmented single-celled photosynthetic eukaryotes, which can be found in almost every type of aquatic environment around the globe (Kociolek 2007, Pla et al. 2016). These microalgae are characterised by an outer silica wall (frustule) that makes them easy to collect and preserve for later identification. Benthic diatoms, in particular, are important contributors to primary production in streams and are widely used as indicators for monitoring the ecological status of aquatic systems and also for past environmental and climatic reconstructions (Battarbee et al. 2006), since many species have distinct ecological optima and narrow tolerance (Cohen 2003). Diatom communities inhabiting streams have been studied in several regions of the globe (e.g. Tison et al. 2005, Grenier et al. 2006, Mora et al. 2017, Falasco et al. 2016, Liu et al. 2019), including insular streams (Delgado et al. 2012, Delgado et al. 2013, Kopalová et al. 2011, Kopalová and van de Vijver 2013, Gonçalves et al. 2015, Vasselon et al. 2017).

Despite their great importance, current knowledge about the freshwater diatoms on insular streams of Madeira Archipelago is limited in comparison with freshwater macroinvertebrates (Hughes et al. 1998, Hughes and Furse 2001, Hughes 2006), bryophytes (Sérgio and Fontinha 1994, Sérgio et al. 2006, Sim-Sim et al. 2008, Sim-Sim et al. 2014, Sim-Sim et al. 2011, Boch et al. 2019) and hyphomycetes communities (Raposeiro et al. 2020). Although diatoms from the Madeira Archipelago have been a matter of study for more than 150 years (Grunow 1867, De-Toni 1891, De-Toni 1892, Zimmermann 1909, Zimmermann 1911, Schodduyn 1927, Mölder 1947, Lange-Bertalot 1993, Kaufmann et al. 2015, Gonçalves et al. 2016), including the description of two regional endemisms, little is known about the regional overall diversity of these microalgae in Madeira Island. The importance of insular freshwater studies of microalgal diversity is centred around the concept that these ecosystems tend to be less complex, providing much potential for testing ideas about biogeographic theory and species distribution limits (Flower 2005).

Here, we provide a detailed dataset that contains freshwater diatom occurrences collected during a field campaign on Madeira Island, increasing the knowledge on the epilithic diatom inhabiting permanent streams in Madeira Island. Our purpose is to release this valuable dataset, since no similar datasets have been previously published for Madeira Archipelago and it constitutes a relevant tool of comparison for aquatic ecologists, for example, biogeographic patterns, climate change or other studies on oceanic islands.

## Project description

### Title

Diatom diversity and distribution in Madeira Island streams (Portugal)

### Personnel

Collections were undertaken and occurrence data recorded during the spring of 2015 in Madeira Island. The collectors were Pedro Raposeiro and Vitor Gonçalves. Identifications were made by Catarina Ritter and supervised by Vitor Gonçalves.

### Study area description

**Figure 1.**
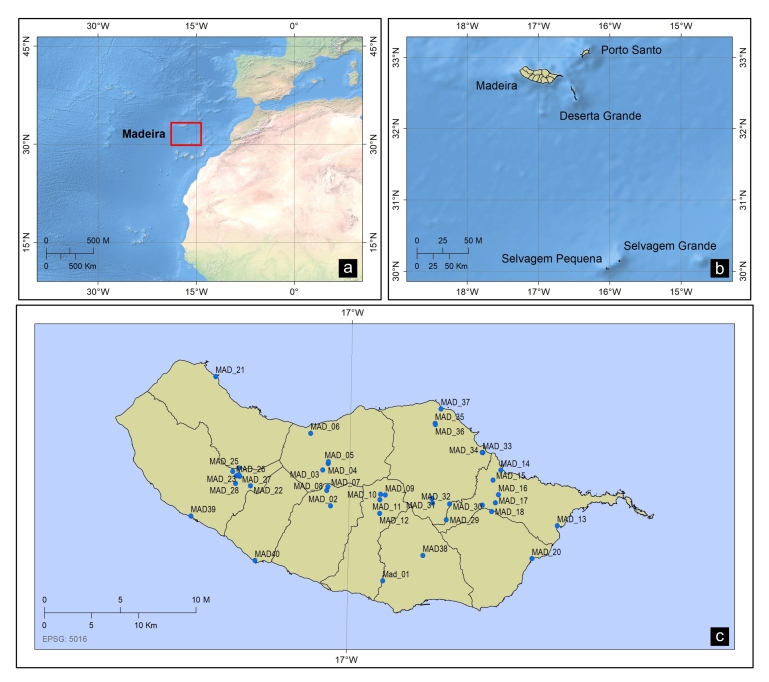
Geographical location of the study stream sites. **a.** Madeira Archipelago in the Atlantic Ocean highlighted by a red square; **b.** Madeira Island in the Madeira Archipelago; **c.** Studied stream sites.

The Madeira Archipelago is an oceanic archipelago located in the North Atlantic between latitudes 32°24' and 33°07'N and longitudes 16°16' and 17°16'W (Fig. 1). Madeira Island is the highest (Pico Ruivo - 1861 m) and largest island (~ 740 km^2^) of the archipelago and about 90% of its area is higher than 500 m above sea level (Ribeiro 1985). Madeira Island presents a high diversity of habitat types, including the largest surviving area of Laurissilva forest in Macaronesia, classified as a UNESCO World Natural Heritage site (IUCN 1999). Due to its oceanic condition, Madeira Island presents a mild temperate oceanic climate strongly influenced by winds from the NE and the Canary Islands current, presenting a relative humidity between 55-75% and annual rainfall between 500 and 1,000 mm (AEMET & IM 2012). An important aspect of the climate in Madeira Island is the persistent nebulous covering of fog, which normally exists in high altitude resulting in an important source of groundwater recharge (Prada et al. 2005). Under this mild temperate oceanic climate, groundwater hydrology is essential for surface water and for the persistence and functioning of the insular aquatic ecosystems as a high number of the permanent streams are fed by springs.

Madeira Island comprises approximately 126 catchments and 200 streams presenting a typical radial drainage pattern common in oceanic islands (Marques 1994). According to Prada et al. 2005, the hydrographic network present in the Island is characterised by deep narrow valleys with a typical U-transverse profile as these are still in a young phase. Most of the streams have a torrential character with high flow rates (Hughes 2006).

## Sampling methods

### Study extent

Epilithic freshwater diatoms (Bacillariophyta Karsten 1928) from 40 sites (MAD01 – MAD40) from 27 permanent streams in Madeira Island.

### Sampling description

**Figure 2.**
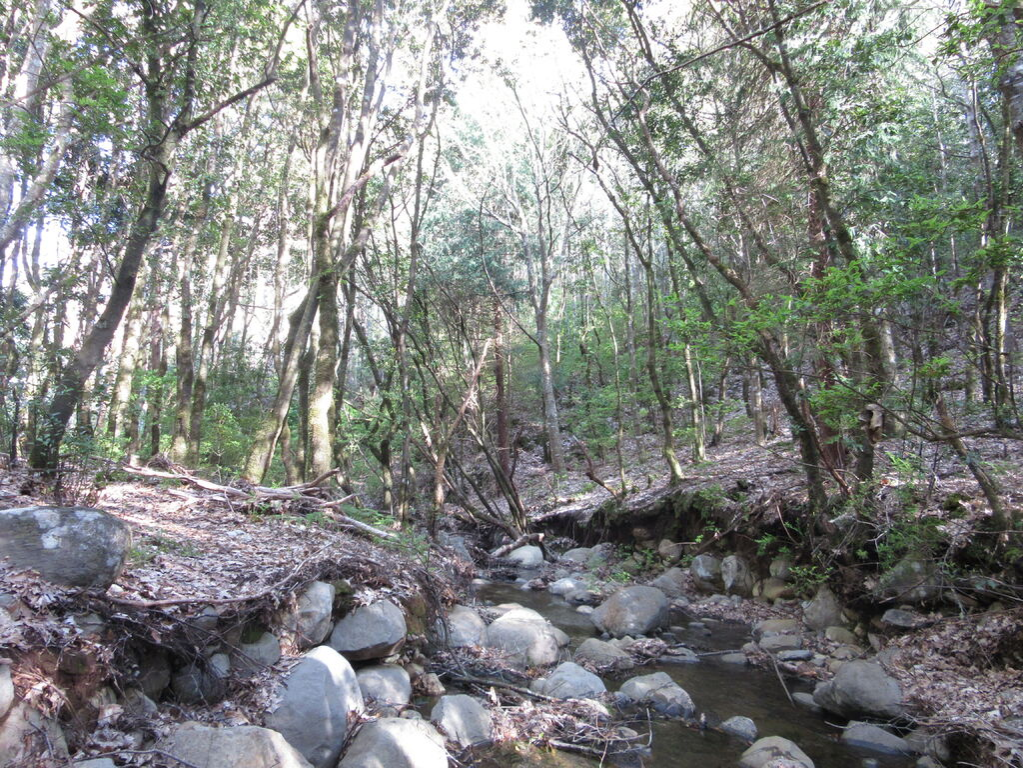
Sampling site representing the natural land-use. Located at Ribeira Primeira, Santo António da Serra, Machico (MAD18).

**Figure 3.**
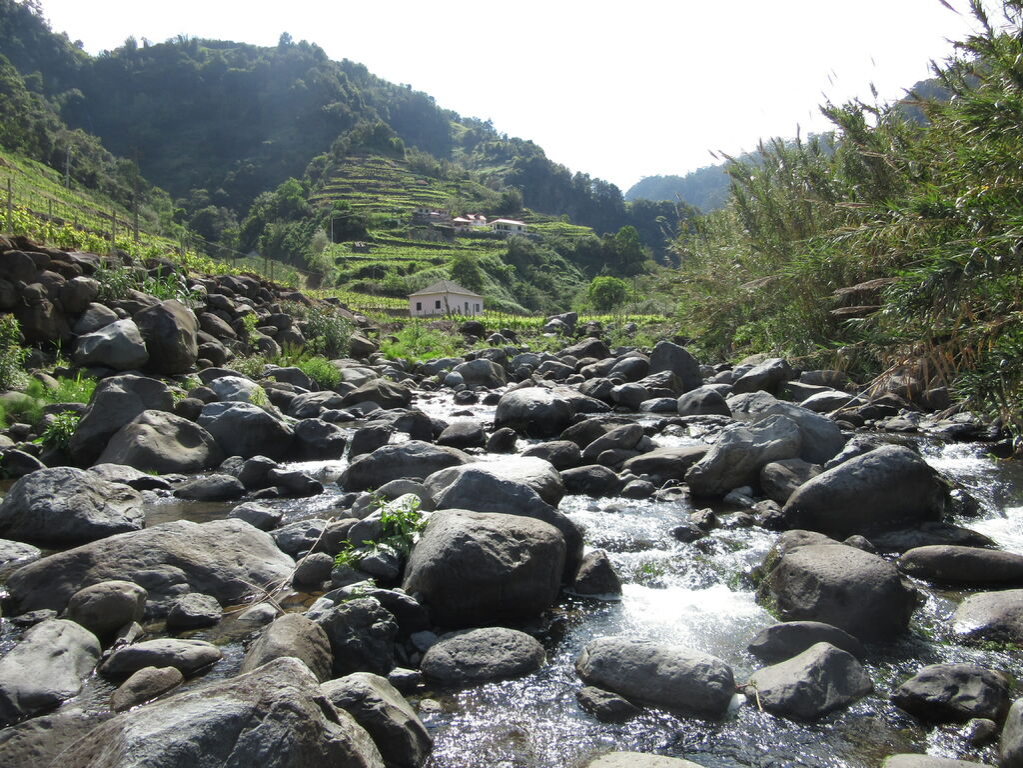
Sampling site representing the agricultural land-use. Located at Ribeira de São Jorge, Santana (MAD37).

**Figure 4.**
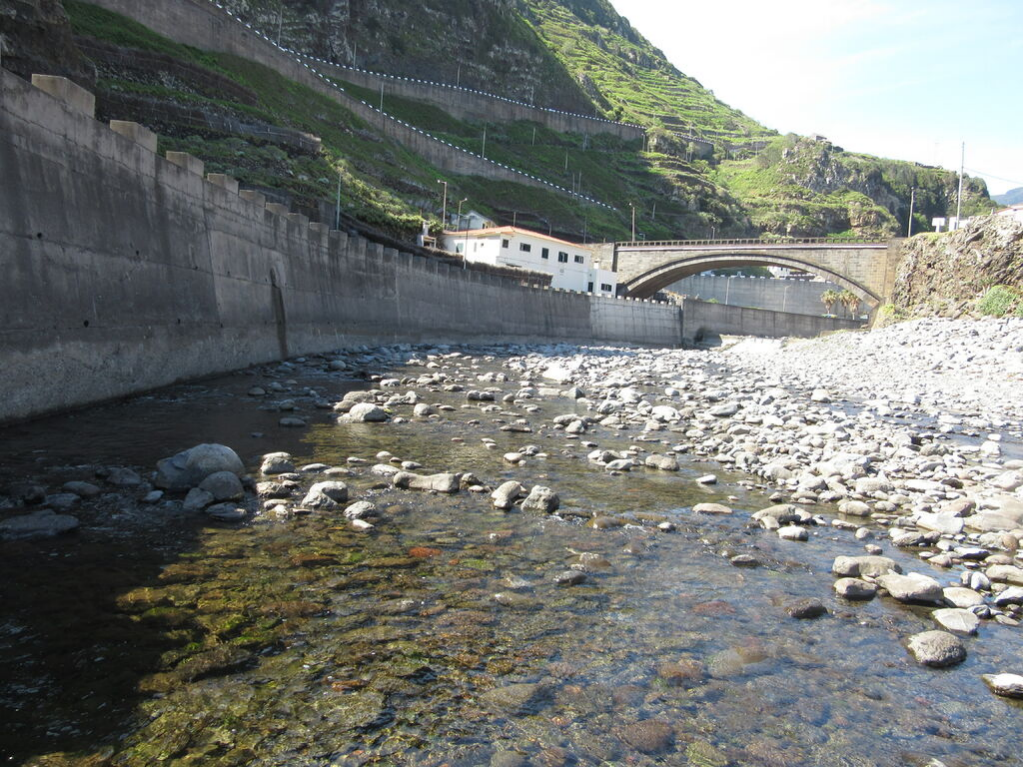
Sampling site representing an urban land-use. Located at Ribeira da Janela, Porto Moniz (MAD21).

**Figure 5.**
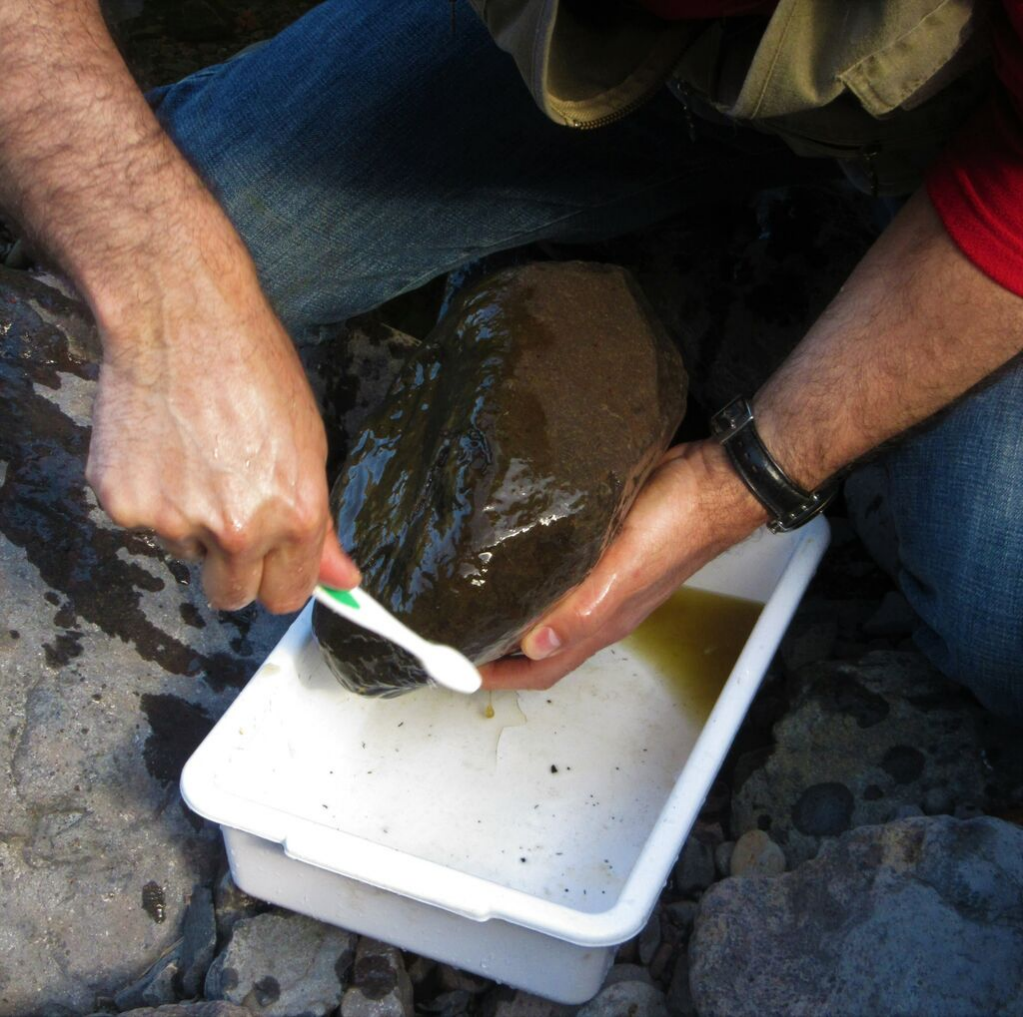
Collecting epilithic diatoms from stones with a toothbrush at each sampling site.

In the spring of 2015, epilithic biofilm samples were collected in 40 sites (MAD01 – MAD40) from 27 permanent streams in Madeira Island (Table 1). The sampling sites ranged in altitudes (low, medium and high) and land-uses (natural, agricultural and urban) (Figs 2, 3, 4). For diatom analysis, samples were prepared following the European (Kelly et al. 1998, European Committee for Standardization 2003, European Committee for Standardization 2004) and national recommendations (INAG 2008). Epilithic diatoms were taken from stones with a toothbrush in each sampling site (Fig. 5). Immediately after collection, diatom samples were fixed with formalin at 4% final concentration. Permanent slides were prepared with Naphrax® and at least 400 valves per sample were counted and identified at the lowest taxonomic level possible under oil-immersion phase contrast light microscopy using a Leica DM2500 (Leica Microsystems GmbH, Welzlar, Germany).

### Quality control

Diatom morphometric features were determined by photomicrography (Leica DFC495) with the aid of image analysis software (LAS version 3.8.0). Diatom identification was based on reference diatom floras (e.g. Krammer and Lange-Bertalot 1986, Krammer and Lange-Bertalot 1988, Krammer and Lange-Bertalot 1991, Krammer and Lange-Bertalot 2000, Krammer and Lange-Bertalot 2002, Lange-Bertalot et al. 2017), as well as on recent bibliographic sources, including the series “Diatoms of Europe”, “Bibliotheca Diatomologica”, relevant taxonomic papers (e.g. Trobajo et al. 2013, Wetzel et al. 2015) and studies in Portuguese freshwater diatoms (Novais et al. 2014). Nomenclatural and taxonomic status used here follows *Algaebase (Guiry and Guiry 2020*).

### Step description

The data have been published as a Darwin Core Archive (DwC-A), which is a standardised format for sharing biodiversity data as a set of one or more data tables. The core data table contains 965 occurrences with 130 records (Ritter et al. 2020).

## Geographic coverage

### Description

Madeira Island, Madeira Archipelago, Macaronesia, Portugal.

### Coordinates

32.6228N and 32.8815N Latitude; -17.2739W and -16.6487W Longitude.

## Taxonomic coverage

### Description

All diatoms were identified to genus or species level. In total, 130 taxa were identified belonging to five subclasses, 17 orders, 27 families and 44 genera distributed in the subphylums Coscinodiscophytina and Bacillariophytina.

### Taxa included

**Table:** 

Rank	Scientific Name	Common Name
phylum	Bacillariophyta	Diatom

## Traits coverage

### Data coverage of traits

PLEASE FILL IN TRAIT INFORMATION HERE

## Usage licence

### Usage licence

Open Data Commons Attribution License

### IP rights notes

This work is licensed under a Creative Commons Attribution (CC-BY) 4.0 License.

## Data resources

### Data package title

Diatom distribution in Madeira Island streams (Portugal)

### Resource link

http://ipt.gbif.pt/ipt/resource?r=occmad&v=1.2

### Alternative identifiers

https://www.gbif.org/dataset/296407fd-6661-4ac8-afa0-f480d5e1d4de

### Number of data sets

1

### Data set 1.

#### Data set name

Diatom distribution in Madeira Island streams (Portugal)

#### Data format

Darwin Core Archive

#### Number of columns

30

#### Data format version

1.2

#### Description

This paper presents data from freshwater diatoms surveys developed in Madeira Island in 2015. The dataset has been published as a Darwin Core Archive (DwC-A), which is a standardised format for sharing biodiversity data as a set of one or more data tables. The core data table contains 40 events (eventID), 965 occurrences (occurrenceID) with 130 taxa (taxonID). The number of records in the data table is illustrated in the IPT link. This IPT archives the data and thus serves as the data repository. The data and resource metadata are available for downloading in the downloads section.

**Data set 1.** 

Column label	Column description
scientificNameAuthorship	The authorship information for the scientificName.
type	The nature of the resource.
basisofRecord	The specific nature of the data record.
occurrenceID	Identifier of the record, coded as a global unique identifier.
eventID	Identifier of the event, unique for the dataset.
eventDate	Time interval when the event occurred.
continent	Continent of the sampling site.
waterBody	Water body of the sampling site.
islandGroup	Island group of the sampling site.
island	Island from the Island Group of the sampling site.
country	Country of the sampling site.
countryCode	Code of the country where the event occurred.
municipality	Name of the municipality where the event occurred.
locality	Name of the locality where the event occurred.
decimalLatitude	The geographic latitude of the sampling site.
decimalLongitude	The geographic longitude of the sampling site.
geodeticDatum	The spatial reference system upon which the geographic coordinates are based.
taxonID	The identifier for the set of taxon information (data associated with the Taxon class). Specific identifier to the dataset.
scientificName	The name with authorship applied on the first identification of the specimen.
acceptedNameUsage	The specimen accepted name, with authorship.
kingdom	Kingdom name.
phylum	Phylum name.
class	Class name.
order	Order name.
family	Family name.
genus	Genus name.
specificEpithet	The name of the first or species epithet of the scientificName.
infraspecificEpithet	The name of the lowest or terminal infraspecific epithet of the scientificName, excluding any rank designation.
taxonRank	The taxonomic rank of the most specific name in the scientificName.
coordinateUncertaintyInMetres	The indicator for the accuracy of the coordinate location in metres, described as the radius of a circle around the stated point location.

## Additional information

### Analysis

This study presents 965 diatom (Bacillariophyta) occurrences in 40 sites in Madeira Island, belonging to 130 different taxa from 44 genera, 27 families, 4 suborders, 17 orders, 5 subclasses, 3 classes and 2 subphyllums (Table 2).

The subphylum Coscinodiscophytina, represented by one class, two orders and three families, accounted for 1.5% of the total occurrences, while the subphylum Bacillariophytina registered 98.4% of the total occurrences. With two classes and four subclasses, most occurrences (815) were registered in the Bacillariophycidae subclass.

The families with the highest number of occurrences were Bacillariaceae (176), Achnanthidiaceae (135) and Naviculaceae (133). However, the families with the highest number of taxa were Bacillariaceae (23), Naviculaceae (15), Achnanthidiaceae (15) and Rhoicospheniaceae (10). The families with lower occurrences (< 5) were Surirellaceae (1), Diploneidaceae (2), Aulacoseiraceae (2), Eupodiscaceae (2) and Stephanodiscaceae (2). However, the families with the smallest number of diatom taxa were Eupodiscaceae (1), Naviculales incertaesedis (1), Surirellaceae (1) and Ulnariaceae (1).

The genera with the highest number of occurrences were *Nitzschia* (174), *Navicula* (130), *Planothidium* (73), *Cocconeis* (54), *Sellaphora* (51), *Fragilaria* (51) and *Gomphonema* (50). The other 37 genera had less than 50 occurrences. The genera with the highest number of taxa were *Nitzschia* (22) and *Navicula* (13).

*Achnanthidium minutissimum* (Kützing) Czarnecki and *Planothidium lanceolatum* (Brébisson ex Kützing) Lange-Bertalot were the most frequent species occurring in 38 from 40 sites (MAD20 and MAD13 were the exceptions). *Nitzschia soratensis* E.A.Morales & M.L.Vis (37 sites), Cocconeis placentula var. euglypta (Ehrenberg) Grunow (36 sites), *Amphora pediculus* (Kützing) Grunow ex A.Schmidt (34 sites), *Navicula reichardtiana* Lange-Bertalot (33 sites) and *Ulnaria ulna* (Nitzsch) Compère (31 sites) were amongst the most ubiquitous diatoms.

A total of 45 diatom taxa occurring at only one sampling site were considered rare. These include benthic species, such as *Achnanthes minuscula* Hustedt, *Achnanthidium pyrenaicum* (Hustedt) H.Kobayasi, *Adlafia minuscula* (Grunow) Lange-Bertalot, *Adlafia multnomahii* E.A.Morales & M.Lee, *Caloneis silicula* (Ehrenberg) Cleve, *Encyonema amanianum* Krammer, *Eunotia arcus* Ehrenberg, *Eunotia paludosa* Grunow, *Frustulia vulgaris* (Thwaites) De Toni, *Gomphonema augur* Ehrenberg, *Luticola mutica* (Kützing) D.G.Mann, *Nitzschia brevissima* Grunow, *Nitzschia filiformis* (W.Smith) Van Heurck, *Planothidium daui* (Foged) Lange-Bertalot, *Rhopalodia gibba* (Ehrenberg) Otto Müller, amongst others; planktonic species, such as *Aulacoseira granulata* (Ehrenberg) Simonsen; tychoplanktonic species, such as *Fragilaria capucina* Desmazières, *Pseudostaurosira elliptica* (Schumann) Edlund, Morales & Spaulding, *Pseudostaurosira robusta* (Fusey) D.M.Williams & Round, *Pseudostaurosira subconstricta* (Grunow) Kulikovskiy & Genkal, *Staurosirella lapponica* (Grunow) D.M.Williams & Round, *Stephanodiscus minutulus* (Kützing) Cleve & Möller, *Stephanodiscus parvus* Stoermer & Håkansson; and aerophilic species, such as *Diploneis elliptica* (Kützing) Cleve, *Diploneis praetermissa* Lange-Bertalot & A.Fuhrmann, *Humidophila brekkaensis* (Petersen) R.L.Lowe, Kociolek, J.R.Johansen, Van de Vijver, Lange-Bertalot & Kopalová and *Orthoseira roeseana* (Rabenhorst) Pfitzer.

Another 32 diatom taxa were considered occasional, occurring in two to five sampling sites. These included benthic species, such as *Achnanthidium gracillimum* (F.Meister) Lange-Bertalot, *Amphora inariensis* Krammer, *Craticula molestiformis* (Hustedt) Mayama, *Eunotia implicata* Nörpel, Lange-Bertalot & Alles, *Gomphonema acuminatum* Ehrenberg, *Gomphosphenia lingulatiformis* (Lange-Bertalot & E.Reichardt) Lange-Bertalot, *Grunowia solgensis* (A.Cleve) Aboal, *Hippodonta capitata* (Ehrenberg) Lange-Bertalot, Metzeltin & Witkowski, *Luticola goeppertiana* (Bleisch) D.G.Mann ex J.Rarick, S.Wu, S.S.Lee & Edlund, *Navicula madeirensis* Lange-Bertalot, *Navicula recens* (Lange-Bertalot) Lange-Bertalot, *Nitzschia recta* Hantzsch ex Rabenhorst, *Planothidium amphibium* C.E.Wetzel, L.Ector & L.Pfister, *Platessa oblongella* (Østrup) C.E.Wetzel, Lange-Bertalot & Ector, *Pleurosira laevis* (Ehrenberg) Compère, *Psammothidium hustedtii* (Krasske) S.Mayama, planktonic species, such as *Fragilaria gracilis* Østrup and the tychoplanktonic species *Staurosirella pinnata* (Ehrenberg) D.M.Williams & Round and *Tabellaria flocculosa* (Roth) Kützing.

The mean number of taxa per sample was 24.1 ± 1.1 SE taxa. MAD40, MAD14, MAD26 and MAD23 were the samples with the highest number of taxa, 49, 38, 34 and 32, respectively. The samples with the lowest number of taxa were MAD20, MAD19 and MAD09 with 11, 14 and 15 taxa, respectively.

The two diatom endemisms, described previously in Madeira Island (Lange-Bertalot, 1993), *Nitzschia macaronesica* Lange-Bertalot and *Navicula madeirensis* Lange-Bertalot, were only observed in a small number of sites. *Nitzschia macaronesica* was present in 10 sites: Ribeira do Cidrão (MAD12), Ribeira do Juncal (MAD14), Corgo da Ribeira dos Aneis (MAD16), Ribeira da Janela (MAD23, MAD24 and MAD26), Ribeira dos Cedros (MAD25), Ribeira da Metade (MAD31), Ribeira das Lages (MAD32) and Ribeira de São Jorge (MAD37). This endemism appeared in sites with a richer diatom community (mean number of taxa of 28.9 ± 1.5 SE), located mostly at high altitude and was associated with *Navicula cryptocephala*, *Navicula reichardtiana*, *Nitzschia soratensis* and *Ulnaria ulna*, apart from the ubiquitous *Achnanthidium minutissimum*, *Amphora pediculus*, Cocconeis placentula var. euglypta and *Planothidium lanceolatum*.

*Navicula madeirensis* occurred only in four sites from different permanent streams: Ribeira do Faial (MAD16), Ribeira do Machico (MAD19), Ribeira do Alecrim (MAD28) and Ribeira da Fonte do Bugio (MAD39). These four sites were distributed in low (1), medium (1) and high (2) altitudes and they have a mean number of taxa below average (23.5 ± 2.9 SE). *Navicula madeirensis* occurred in association with *Planothidium frequentissimum* (Lange-Bertalot) Lange-Bertalot, *Rhoicosphenia abbreviata* (C.Agardh) Lange-Bertalot and *Karayevia clevei* (Grunow) Round & Bukhtiyarova, apart from the ubiquitous *Achnanthidium minutissimum*, *Amphora pediculus*, Cocconeis placentula var. euglypta and *Planothidium lanceolatum*.

In this survey, 60 records were new, not only to Madeira Island, but also to the Madeira Archipelago (Table 3). These include 55 species, two varieties and three genera (sp.).

### Discussion

The diatom diversity (130 taxa belonging to 44 genera) displayed by Madeira Island in the 27 permanent streams in this study is due to the habitat complexity (including water quality, habitat structure and climate), as well as large scale-effects stemming from the Islands’ isolation and geographical location as was found on other oceanic islands (Flower 2005, Gonçalves et al. 2015).

Diatom diversity from Madeira Island is relatively low when compared to other oceanic islands and continental regions (e.g. Herlory et al. 2013, Gonçalves et al. 2015, Jyrkänkallio-Mikkola et al. 2018). This kind of comparison is difficult to make since it depends on the sampling efforts: the number of samples analysed, the timing of the samplings, the number of surveys carried out, the physical and chemical composition of the waters, the number of substrates sampled and the taxonomic effort with which the diatom valves were analysed (Morales et al. 2009, Veselá and Johansen 2009).

Nonetheless, when comparing archipelagos in the Macaronesia Region, 201 diatom taxa were recorded in 316 samples from 14 permanent Azorean streams (Gonçalves et al. 2015). However, the number of diatom taxa recorded per sample was higher in Madeira Island (24.1 ± 1.1 SE) than in the Azores (20.9 ± 2.7 SE). Additionally, it is worth mentioning that this study is focused on the main island from the Madeira Archipelago which does not represent the different microhabitats present in all the other islands.

Comparisons to other regions in the world reveal how diatom diversity in Madeira Island is “poor”. For instance, in a tropic region (Sub-Saharan Africa), the number of diatom taxa identified in 67 sites in Kenya was significantly greater (297 taxa) than the number of taxa recorded in Madeira Island (Jyrkänkallio-Mikkola et al. 2018).

The low diversity of freshwater biota has already been reported to the Madeira Archipelago (Hughes and Malmqvist 2005), as well as other oceanic islands in the world (Brasher et al. 2004, Flower 2005, Delgado et al. 2012).

This insular oceanic ecosystem should offer some degree of isolation from continental floras, but the special conditions that promote speciation on islands are not present, for example, extreme water quality or geological age and activity (Flower 2005). It is therefore unsurprising that the great majority of taxa had a cosmopolitan distribution (e.g. *Achnanthidium minutissimum*, *Planothidium lanceolatum*, *Nitzschia soratensis*, Cocconeis placentula var. euglypta) (Flower 2005, Gonçalves et al. 2015). This indicates a lack of isolation mechanisms operating in the Island, but the diatom taxa present are nevertheless clearly attributable to different biogeographical regions.

The Macaronesia Region should register more endemisms for many groups as do other regions (e.g. Antarctic Region, Van de Vijver et al. 2011, Van De Vijver et al. 2004, Kopalová et al. 2009, Kopalová et al. 2011), but due to the lack of research in this Region as stated before, only a few have been found. This might be one of the reasons why we had some taxonomic challenges. Diatoms are extremely diverse and there are many species that have not been described yet, thus the species delimitation is still controversial (Licea et al. 2016). For some diatom groups, it was difficult to distinguish closely-related species because of their wide morphological variation or because many taxa frequently differed in detail from published descriptions. We took a conservative attitude for these taxonomic differences and disregarded small differences in morphometric data.

Considering the significant differences between the islands of Madeira Archipelago, such as geological ages, volcanic composition, climate patterns and distribution, land uses, types of forest and orography, we expect higher diatom species richness and exclusive taxa from these islands (Porto Santo, Desertas, Selvagens). Furthermore, increasing the sampling effort in Madeira Island, for instance, by sampling other streams and/or other sites in the Laurissilva forest, may result in the identification of other diatom taxa. Additionally, higher time replication and larger datasets are required to better understand distribution patterns and large-scale spatial patterns of species dispersal. According to Smucker and Vis (2011), there is a significant under-estimation of diatom diversity when exclusively collecting epilithic habitats for documenting species distribution and for conservation purposes. Taking this into consideration, different methods to obtain the greater number of diatom taxa for each site are encouraged, for instance, by following two distinct sampling techniques (transects located in riffles and from microhabitats) (Falasco et al. 2016).

The factors controlling taxa richness, as well as the regional endemic taxa, remain unclear as there is no convincing link with the simplified habitat features recorded during the study and more research is needed. Relationships are probably multivariate in nature and include site history, unmeasured micro-habitat availability and climate (Flower 2005, Pajunen et al. 2016).

The results of this study provide baseline knowledge on the current distribution of freshwater diatoms on Madeira Island streams, revealing a distinct, but taxonomically simple diatom flora, typical from oceanic island ecosystems (Flower 2005, Gonçalves et al. 2015). In order to better understand the complexity of these streams, depth studies on the temporal and spatial distribution patterns, population dynamics, species’ interactions, guilds and traits are essential for improving knowledge and the development of future effective monitoring and conservation programmes and measures for local stakeholders.

## Figures and Tables

**Table 1.** Sampling codes and location of the forty studied stream sites on Madeira Island.

**Sampling code**	**Stream**	**Municipality**	**Sampling date**	**Latitude(ºN) / Longitude(ºW)**	**Altitude(m)**
MAD01	Ribeira dos Socorridos	Câmara de Lobos	28/04/2015	32.66319, -16.9606	85
MAD02	Ribeira Brava	Ribeira brava	28/04/2015	32.73395, -17.021	409
MAD03	Ribeira da Vargem	São Vicente	28/04/2015	32.76807, -17.0305	450
MAD04	Ribeira de São Vicente	São Vicente	28/04/2015	32.77415, -17.0245	325
MAD05	Ribeira Grande	São Vicente	28/04/2015	32.77599, -17.0244	311
MAD06	Ribeira Grande	São Vicente	28/04/2015	32.28433, -16.7232	60
MAD07	Ribeira Brava	São Vicente	28/04/2015	32.75216, -17.0244	903
MAD08	Ribeira Brava	São Vicente	28/04/2015	32.74842, -17.0257	833
MAD09	Ribeira dos Socorridos	Câmara de Lobos	29/04/2015	32.74522, -16.9591	826
MAD10	Ribeira da Gomeira	Câmara de Lobos	29/04/2015	32.74572, -16.9646	725
MAD11	Corgo da Ribeira de Anéis	Câmara de Lobos	29/04/2015	32.74059, -16.9652	780
MAD12	Ribeira do Cidrão	Câmara de Lobos	29/04/2015	32.72749, -16.9653	597
MAD13	Ribeira do Machico	Machico	29/04/2015	32.71876, -16.7642	10
MAD14	Ribeira do Juncal	Machico	29/04/2015	32.77081, -16.8289	36
MAD15	Ribeira do Juncal	Machico	29/04/2015	32.76142, -16.8376	187
MAD16	Ribeira do Faial	Machico	29/04/2015	32.74741, -16.8313	560
MAD17	Ribeira do Machico	Machico	29/04/2015	32.73962, -16.8347	624
MAD18	Ribeira Primeira	Machico	29/04/2015	32.73101, -16.8388	791
MAD19	Ribeira do Machico	Machico	29/04/2015	32.73715, -16.8493	877
MAD20	Ribeira de Santa Cruz	Santa Cruz	29/04/2015	32.68695, -16.792	7
MAD21	Ribeira da Janela	Porto Moniz	30/04/2015	32.85522, -17.1537	81
MAD22	Ribeira do Alecrim	Porto Moniz	30/04/2015	32.75164, -17.1121	1391
MAD23	Ribeira da Janela	Porto Moniz	30/04/2015	32.7603, -17.1241	1089
MAD24	Ribeira da Janela	Porto Moniz	30/04/2015	32.76077, -17.1283	1041
MAD25	Ribeira dos Cedros	Porto Moniz	30/04/2015	32.76582, -17.1256	899
MAD26	Ribeira da Janela	Porto Moniz	30/04/2015	32.76503, -17.1324	1003
MAD27	Ribeira da Janela	Porto Moniz	30/04/2015	32.76191, -17.1252	1271
MAD28	Ribeira do Alecrim	Porto Moniz	30/04/2015	32.7535, -17.129	1182
MAD29	Ribeira Frio	Santana	01/05/2015	32.72254, -16.8897	846
MAD30	Córrego do Arrochete	Santana	01/05/2015	32.73768, -16.8864	637
MAD31	Ribeira da Metade	Santana	01/05/2015	32.74293, -16.9064	686
MAD32	Ribeira das Lajes	Santana	01/05/2015	32.73838, -16.9057	23
MAD33	Ribeira de São Roque do Faial	Santana	01/05/2015	32.78725, -16.8497	42
MAD34	Ribeira Seca	Santana	01/05/2015	32.78758, -16.8505	103
MAD35	Ribeira de São Jorge	Santana	01/05/2015	32.81442, -16.9044	121
MAD36	Ribeira dos Arcos	Santana	01/05/2015	32.81342, -16.904	517
MAD37	Ribeira de São Jorge	Santana	01/05/2015	32.82849, -16.8978	21
MAD38	Ribeira de Santa Luzia	Funchal	02/05/2015	32.67818, -16.9182	25
MAD39	Ribeira da Fonte do Bugio	Calheta	02/05/2015	32.72153, -17.1784	22
MAD40	Ribeira da Ponta do Sol	Ponta do Sol	02/05/2015	32.6803, -17.1052	85

**Table 2.** Main taxonomic figures.

**Orders**	**Families**	**Genera**	**Total taxa**	**Total species**	**New records**	**Madeira endemisms**
Aulacoseirales	1	1	2	1	1	
Bacillariales	1	2	22	21	10	1
Cocconeidales	2	6	18	16	9	
Cymbellales	3	7	18	18	8	
Eunotiales	1	1	4	3	3	
Eupodiscales	1	1	1	1		
Fragilariales	2	3	14	14	9	
Licmophorales	1	1	1	1		
Mastogloiales	1	2	3	2	3	
Melosirales	2	2	2	2		
Naviculales	7	12	2	31	13	1
Rhopalodiales	1	1	31	2		
Stephanodiscales	1	1	2	2	2	
Surirellales	1	1	1	1	1	
Tabellariales	1	2	3	3		
Thalassiophysales	1	1	4	3	1	

**Table 3.** New records for Madeira Archipelago and respective sampling sites.

**Class**	**Order**	**Family**	**First records for Madeira Archipelago**	**Sampling sites**
Bacillariophyceae	Bacillariales	Bacillariaceae	*Nitzschia acidoclinata* Lange-Bertalot 1976	MAD40
			*Nitzschia alpinobacillum* Lange-Bertalot 1993	MAD40
			*Nitzschia clausii* Hantzsch 1860	MAD25
			*Nitzschia dealpina* Lange-Bertalot & Hoffmann 1993	MAD28
			*Nitzschia filiformis* (W.Smith) Van Heurck 1896	MAD14
			Nitzschia filiformis var. conferta (P.G.Richter) Lange-Bertalot 1987	MAD14
			*Nitzschia fonticola* (Grunow) Grunow 1881	MAD06; MAD11; MAD12; MAD13; MAD14; MAD31; MAD34; MAD36; MAD37; MAD40
			*Nitzschia perminuta* Grunow 1881	MAD11; MAD12; MAD14; MAD16; MAD25; MAD34; MAD37; MAD38
			*Nitzschia pusilla* Grunow 1862	MAD08; MAD09; MAD11; MAD21; MAD38
			*Nitzschia recta* Hantzsch ex Rabenhorst 1862	MAD25; MAD26
			*Nitzschia tubicola* Grunow 1880	MAD39
	Cocconeidales	Achnanthidiaceae	*Achnanthidium gracillimum* (F.Meister) Lange-Bertalot 2004	MAD22
			*Achnanthidium jackii* Rabenhorst 1861	MAD30
			*Achnanthidium saprophilum* (H.Kobayashi & Mayama) Round & Bukhtiyarova 1996	MAD32
			*Achnanthidium straubianum* (Lange-Bertalot) Lange-Bertalot 1999	MAD01; MAD33; MAD36
			*Planothidium amphibium* C.E.Wetzel, L.Ector & L.Pfister 2014	MAD09; MAD10; MAD12; MAD21
			*Planothidium daui* (Foged) Lange-Bertalot 1999	MAD19
			*Planothidium dubium* (Grunow) Round & Bukhtiyarova 1996	MAD40
			*Planothidium pumilum* Bąk & Lange-Bertalot 2015	MAD01; MAD34; MAD36; MAD40
			*Psammothidium hustedtii* (Krasske) S.Mayama 2002	MAD17; MAD19; MAD30; MAD35
	Cymbellales	Anomoeoneidaceae	*Adlafia bryophila* (J.B.Petersen) Lange-Bertalot 1998	MAD14; MAD15; MAD21; MAD22; MAD23; MAD36
			*Adlafia minuscula* (Grunow) Lange-Bertalot 1999	MAD21
			*Adlafia multnomahii* E.A.Morales & M.Lee 2005	MAD14
		Gomphonemataceae	*Encyonema amanianum* Krammer 1997	MAD23
			*Gomphonema augur* Ehrenberg 1841	MAD40
			*Gomphonema clavatulum* E.Reichardt 1999	MAD07; MAD08; MAD11; MAD14; MAD15; MAD23; MAD25; MAD26; MAD27; MAD31
			*Gomphonema minutum* (C.Agardh) C.Agardh 1831	MAD23; MAD40
		Rhoicospheniaceae	*Gomphosphenia lingulatiformis* (Lange-Bertalot & E.Reichardt) Lange-Bertalot 1991	MAD14; MAD15; MAD39
	Eunotiales	Eunotiaceae	*Eunotia arcus* Ehrenberg 1837	MAD22
			*Eunotia* sp.	MAD08; MAD18; MAD19; MAD24; MAD27
			*Eunotia paludosa* Grunow 1862	MAD31
	Fragilariales	Fragilariaceae	*Fragilaria microvaucheriae* C.E.Wetzel & Ector 2015	MAD26; MAD35; MAD36; MAD37; MAD40
			*Fragilaria pectinalis* (O.F.Müller) Lyngbye 1819	MAD03; MAD21; MAD23; MAD26; MAD36; MAD40
			*Fragilaria perminuta* (Grunow) Lange-Bertalot 2000	MAD16; MAD25
			*Fragilaria recapitellata* Lange-Bertalot & Metzeltin 2009	MAD06; MAD12; MAD31; MAD32; MAD34; MAD38
			*Fragilaria rumpens* (Kützing) G.W.F.Carlson 1913	MAD05; MAD20; MAD22; MAD23; MAD28; MAD30; MAD35
		Staurosiraceae	*Pseudostaurosira elliptica* (Schumann) Edlund, Morales & Spaulding 2006	MAD08
			*Pseudostaurosira robusta* (Fusey) D.M.Williams & Round 1988	MAD30
			*Pseudostaurosira subconstricta* (Grunow) Kulikovskiy & Genkal 2011	MAD26
			*Staurosirella lapponica* (Grunow) D.M.Williams & Round 1987	MAD23
	Mastogloiales	Achnanthaceae	Achnanthes brevipes var. brevipes Agardh 1824	MAD40
			*Achnanthes minuscula* Hustedt 1945	MAD40
	Naviculales	Diadesmidaceae	*Humidophila brekkaensis* (Petersen) R.L.Lowe, Kociolek, J.R.Johansen, Van de Vijver, Lange-Bertalot & Kopalová 2014	MAD27
		Diploneidaceae	*Diploneis praetermissa* Lange-Bertalot & A.Fuhrmann 2016	MAD19
		Naviculaceae	*Caloneis silicula* (Ehrenberg) Cleve 1894	MAD34
			*Hippodonta capitata* (Ehrenberg) Lange-Bertalot, Metzeltin & Witkowski 1996	MAD02; MAD14
			*Navicula angusta* Grunow 1860	MAD13; MAD14; MAD40
			*Navicula cryptotenelloides* Lange-Bertalot 1993	MAD40
			*Navicula recens* (Lange-Bertalot) Lange-Bertalot 1985	MAD26; MAD29
			*Navicula rostellata* Kützing 1844	MAD28; MAD29; MAD40
			*Navicula tantula* Hustedt 1943	MAD07; MAD08; MAD14; MAD25; MAD26, MAD27, MAD28; MAD33; MAD34; MAD37; MAD39
			*Navicula tenelloides* Hustedt 1937	MAD14; MAD 25; MAD27; MAD34
		Sellaphoraceae	*Sellaphora atomoides* (Grunow) Wetzel & Van de Vijver 2015	MAD02; MAD04; MAD07; MAD08; MAD12; MAD13; MAD15; MAD16; MAD17; MAD18; MAD21; MAD22; MAD23; MAD29; MAD30; MAD33; MAD34; MAD36; MAD38; MAD39; MAD40
		Stauroneidaceae	*Craticula molestiformis* (Hustedt) Mayama 1999	MAD11; MAD12; MAD30
			*Fistulifera saprophila* (Lange-Bertalot & Bonik) Lange-Bertalot 1997	MAD01; MAD02; MAD04; MAD09; MAD10; MAD12; MAD13; MAD14; MAD15; MAD16; MAD17; MAD18; MAD20; MAD21; MAD33; MAD34; MAD35; MAD38; MAD40
	Surirellales	Surirellaceae	*Surirella terricola* Lange-Bertalot & E.Alles 1996	MAD40
	Thalassiophysales	Catenulaceae	*Amphora* sp.	MAD31
Coscinodiscophyceae	Aulacoseirales	Aulacoseiraceae	*Aulacoseira* sp.	MAD04
Mediophyceae	Stephanodiscales	Stephanodiscaceae	*Stephanodiscus minutulus* (Kützing) Cleve & Möller 1882	MAD14
			*Stephanodiscus parvus* Stoermer & Håkansson 1984	MAD40
